# Author Correction: An opioid-gated thalamoaccumbal circuit for the suppression of reward seeking in mice

**DOI:** 10.1038/s41467-023-40431-6

**Published:** 2023-08-07

**Authors:** Kelsey M. Vollmer, Lisa M. Green, Roger I. Grant, Kion T. Winston, Elizabeth M. Doncheck, Christopher W. Bowen, Jacqueline E. Paniccia, Rachel E. Clarke, Annika Tiller, Preston N. Siegler, Bogdan Bordieanu, Benjamin M. Siemsen, Adam R. Denton, Annaka M. Westphal, Thomas C. Jhou, Jennifer A. Rinker, Jacqueline F. McGinty, Michael D. Scofield, James M. Otis

**Affiliations:** 1 Department of Neuroscience, Medical University of South Carolina, Charleston, SC 29425 USA; 2 Anesthesiology and Perioperative Medicine, Medical University of South Carolina, Charleston, SC 29425 USA; 3 Hollings Cancer Center, Medical University of South Carolina, Charleston, SC 29425 USA

**Keywords:** Reward, Motivation

Correction to: *Nature Communications* 10.1038/s41467-022-34517-w, published online 11 November 2022

The original version of this Article contained an error in the “Head-Fixed/Acquisition” section of the Methods, in which the cue length was incorrectly stated as 2s, incorrectly reported a gap in time between cue and sucrose delivery as 1s, instead of being followed immediately by sucrose delivery for 2s duration. The incorrect sentence in the Methods states “A press on the active lever, but not inactive lever, resulted in the presentation of a tone cue (8 kHz, 2 s), followed by a gap in time (trace interval; 1 s), and finally the delivery of a liquid sucrose reward (12.5 μl; 12.5% mixed in tap water).”. This has been corrected to “A press on the active lever, but not inactive lever, resulted in the presentation of a tone cue (8 kHz, 1.6s) followed by the delivery of a liquid sucrose reward (12.5 µl; 12.5% mixed in tap water; 2s delivery duration).”.

This error also affected an experimental schematic in Figure 1B which incorrectly showed the cue (gray) as 2s and the timing interval as 1s. The new version of Figure 1B shows the cue is 1.6s (black) and the sucrose delivery is 2s (blue). The error also affected Figures 1J-L, 1V-X, 4D,G, S1G,J, S2D and S7A-B where the cue and delivery times are now represented as bars instead of as dotted lines.

The error also affected the figure legend for Figure 1, where “TI, trace interval” was included. This phrase has now been removed from the figure legend.

These errors have been corrected in both the PDF and HTML versions of the Article.

### Supplementary information

Updated Supplementary Information

