# Supplementary material for: An opioid-gated thalamoaccumbal circuit for the suppression of reward seeking in mice
Source: Nat Commun. 2022 Nov 11;13:6865. doi: 10.1038/s41467-022-34517-w (PMC9652456; doi:10.1038/s41467-022-34517-w)
Supplement: Supplementary file 1 — Supplementary Information [file 41467_2022_34517_MOESM1_ESM.pdf]

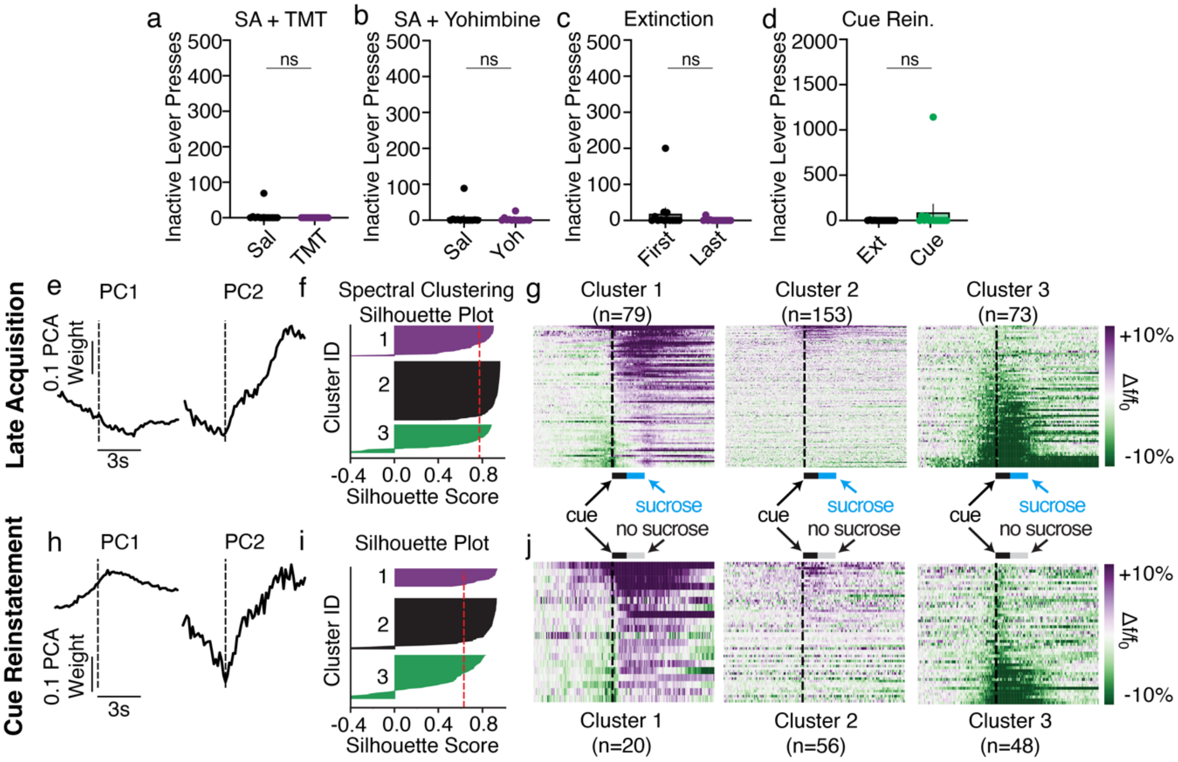

Supplementary Figure 1. Inactive lever responding for *in vivo* imaging, PCA-based spectral clustering silhouette scores and within-cluster response dynamics.

a-d, Inactive lever pressing remained unchanged after TMT exposure (**a**; *n*=13 mice; two-tailed t-test, t_{12}=1.08, *P*=0.30), yohimbine administration (**b**; *n*=13 mice; two-tailed t-test, t_{12}=0.63, *P*=0.54), extinction learning (**c**; *n*=13 mice; two-tailed t-test, t_{12}=1.32, *P*=0.21) and cue-induced reinstatement (**d**; *n*=13 mice; two-tailed t-test, t_{12}=1.09, *P*=0.29; data are presented as mean values +/- SEM). **e, f**, Principal components analysis (**e**) and silhouette plot (**f**) show the relative fit for each neuron for each cluster formed by spectral clustering during late acquisition. **g**, Heatmaps for each neuronal ensemble reveals within-cluster neuronal responses during an active lever press for sucrose. **h, i**, Principal components analysis (**h**) and silhouette plot (**i**) show the relative fit for each neuron for each cluster during cue reinstatement. **j**, Heatmaps for each neuronal ensemble during an active lever press, with no sucrose reward. Rein., Reinstatement. Source data are provided as a Source Data file.

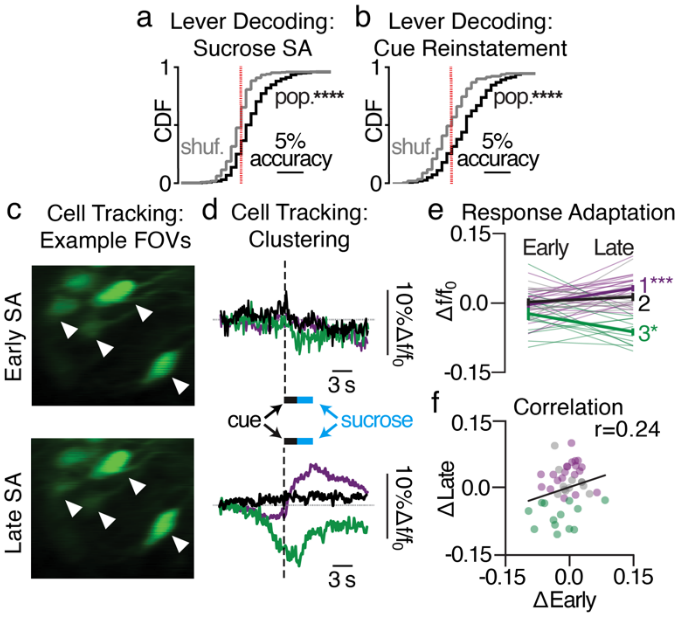

Supplementary Figure 2. Decoding reveals that PVT→NAc population dynamics predict active lever pressing, while cell tracking shows PVT→NAc clusters adapt across learning.

a, CDF plot showing that the activity of all PVT→NAc neurons, not split by cluster, can predict active lever pressing during late acquisition (two-tailed t-test, t_{304}=9.30, ****P<0.001). **b**, CDF plot showing all PVT→NAc neurons, not split by cluster, can predict active lever pressing during cue reinstatement (two-tailed t-test, t_{123}=5.85, ****P<0.001). **c**, Example FOVs for PVT→NAc neurons which were tracked across sucrose self-administration days (n=6 mice, 45 tracked cells); early acquisition day (top), late acquisition day (bottom). **d**, Clustering of the tracked cells across each day revealed a change in ensemble dynamics for excitatory responders (20 cells), non-responders (12 cells), and inhibitory responders (13 cells). **e**, Mean responses for all tracked cells show a significant response adaptation for ensembles 1 and 3 across early and late acquisition days (two-way ANOVA, ensemble x time interaction: $F_{2,84}$=10.08; P<0.001; Sidak's post-hoc: ensembles 1, 3 P-values<0.02). **f**, Correlation plot displaying mean responses (early vs. late) for all tracked neurons (Pearson-R value displayed in top right of graph, P=0.12). SA, self-administration; shuf., shuffled data; pop., population data; FOV, field of view. Group comparisons: *P<0.05, ***P=0.001, ****P<0.001.

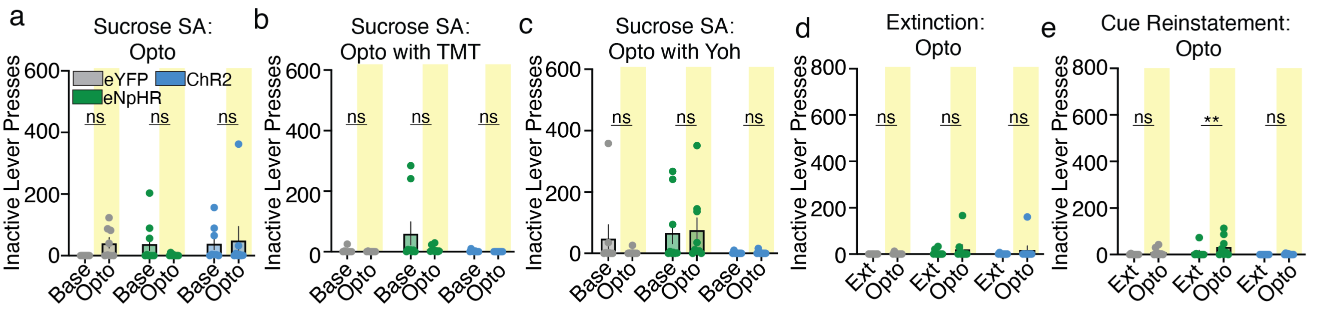

Supplementary Figure 3. Inactive lever pressing was unchanged by optogenetic manipulation of PVT→NAc neurons.

a-e, Inactive lever pressing remained unchanged despite optogenetic manipulation of PVT→NAc neurons for opto (**a**; *n*=8 eYFP, 8 eNpHR, 9 ChR2 mice; repeated-measures two-way ANOVA, *F*-values<1.37, *P*-values>0.28), TMT (**b**; *n*=8 eYFP, 9 eNpHR, 9 ChR2 mice; repeated-measures two-way ANOVA, *F*-values<2.82, *P*-values>0.08), yohimbine (**c**; *n*=8 eYFP, 9 eNpHR, 8 ChR2 mice; repeated-measures two-way ANOVA, group: $F_{2,22}$=3.93, *P*=0.04; post-hoc: *P*-values>0.71), and extinction (**d**; *n*=8 eYFP, 9 eNpHR, 9 ChR2 mice; repeated-measures two-way ANOVA, *F*-values<1.68, *P*-values>0.21). **e**, Optogenetic inhibition of PVT→NAc neurons in eNpHR mice resulted in an increase of inactive lever pressing during cue-reinstatement, though other groups were unchanged (*n*=8 eYFP, 8 eNpHR, 9 ChR2 mice; repeated-measures two-way ANOVA, day: $F_{1,22}$=9.23, *P*=0.006; post-hoc: eNpHR ***P*=0.004). SA, self-administration; Base, baseline; Opto, optogenetics; Yoh, yohimbine. Data are presented as mean values +/- SEM. Source data are provided as a Source Data file.

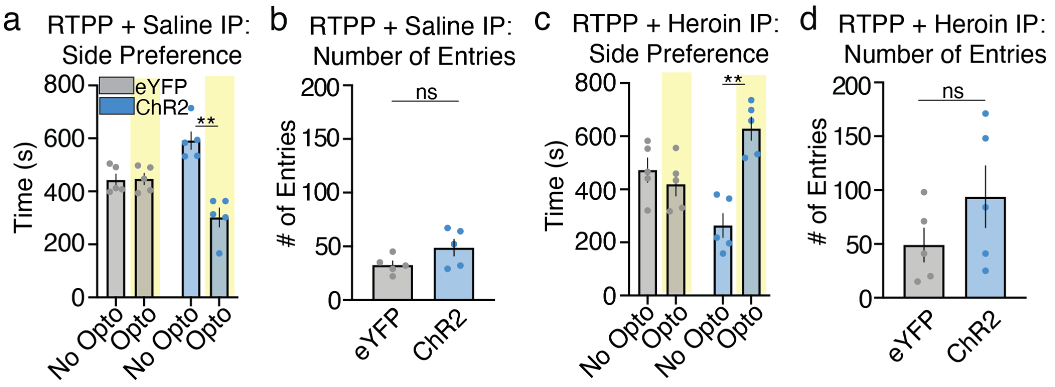

Supplementary Figure 4. Heroin administration causes stimulation of PVT→NAc neurons to be appetitive, rather than aversive.

a, Optogenetic stimulation of PVT→NAc neurons resulted in a real-time place aversion in ChR2 mice, but not eYFP mice (*n*=5 mice/group; repeated-measures two-way ANOVA, side x group: $F_{1,8}$=12.89, *P*=0.007; post-hoc: ChR2 ***P*=0.002, eYFP *P*=0.99). **b**, Optogenetic stimulation of PVT→NAc neurons did not affect locomotion, as there was not a significant difference in the number of chamber entries between eYFP and ChR2 mice (*n*=5 mice/group; two-tailed t-test, t_4=2.59; *P*=0.06). **c**, A single intraperitoneal injection of heroin resulted in stimulation-dependent real-time place preference in ChR2 mice, but not eYFP mice (*n*=5 mice/group; repeated-measures two-way ANOVA, side x group: $F_{1,8}$=10.85, P=0.01; post-hoc: ChR2 ***P*=0.007, eYFP *P*=0.82). **d**, Optogenetic stimulation of PVT→NAc neurons did not affect locomotion in heroin-treated mice, as there was not a significant difference in the number of chamber entries between eYFP and ChR2 mice (*n*=5 mice/group; two-tailed t-test, t_4=2.59; *P*=0.06). RTPP, real-time place preference; IP, intraperitoneal injection; Opto, optogenetics. Data are presented as mean values +/- SEM. Source data are provided as a Source Data file.

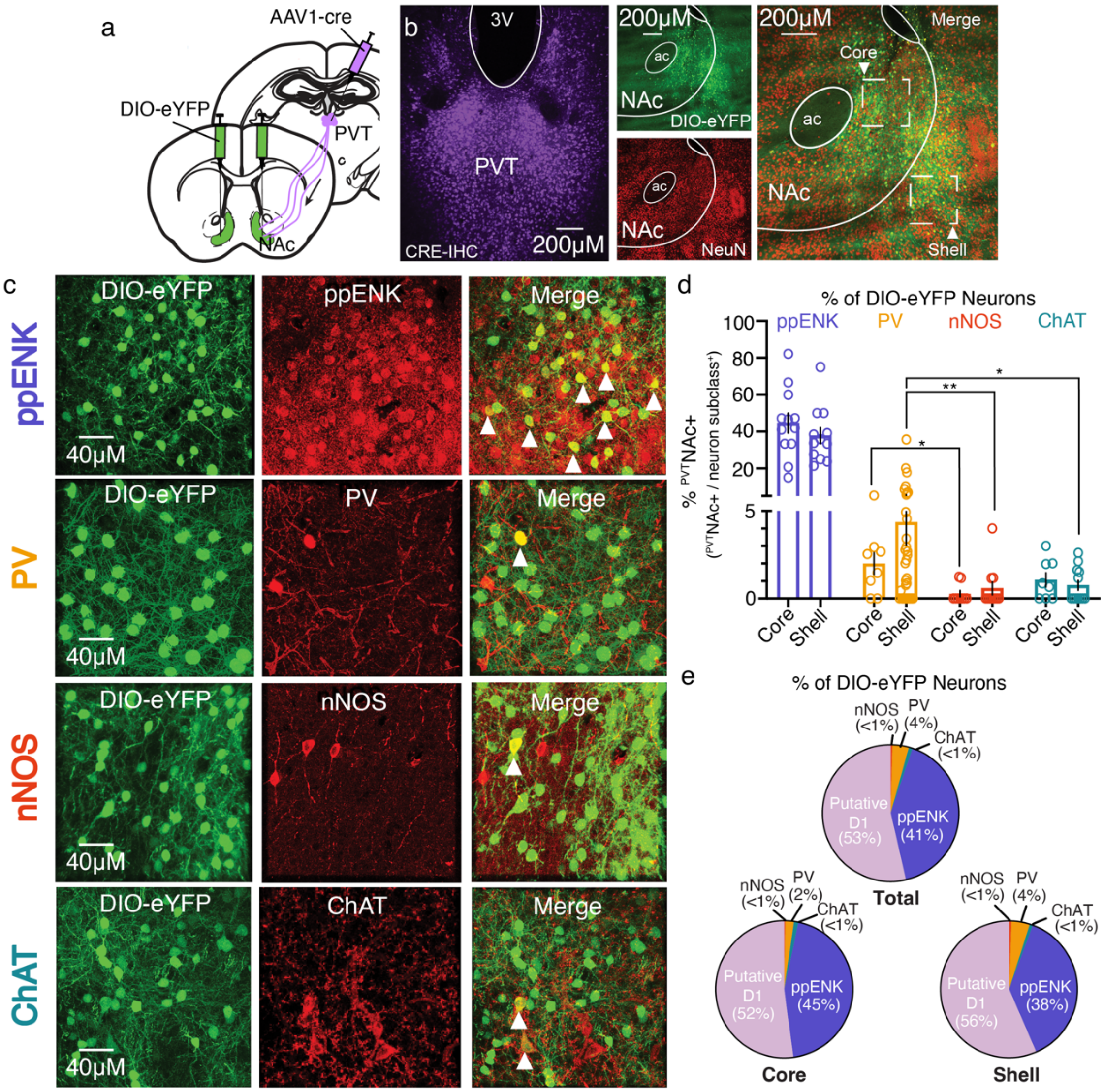

Supplementary Figure 5. Identification of downstream PVT projection targets reveals synaptic innervation of D1-MSNs, D2-MSNs, and PV interneurons.

a, Surgical strategy wherein the anterogradely trafficked virus AAV1-Cre[27] was used to label downstream cellular targets of PVT→NAc projection neurons. **b**, Example immunohistochemistry (IHC) for Cre, showing viral transduction of AAV1-Cre in posterior PVT (left); representative images of DIO-eYFP labeling and NeuN IHC in NAc core and shell (middle); merged image of Cre-inducible eYFP and NeuN in NAc (right). **c**, Representative images showing anterogradely labeled eYFP$^+$ neurons (left; neurons receiving strong PVT input), IHC for ppENK (putative D2 MSNs), PV, nNOS, and ChAT (middle), and overlayed images (right). **d**, Percentage of co-labeled eYFP$^+$ and neuron subclass markers in neurons of the NAc core versus shell. Anterograde PV interneuron labeling was elevated compared to other striatal interneurons in NAc shell (*n*=3 mice, 68 cells; one-way ANOVA, effect of cell type: $F_{3,64}$=61.39, P<0.001; Sidak's post-hoc: PV vs nNOS **P=0.008; PV vs ChAT *P=0.01), but only greater than one striatal interneuron subtype in NAc core (*n*=3 mice, 37 cells; one-way ANOVA, effect of cell type: $F_{3,33}$=41.42, P<0.001; Sidak's post-hoc: PV vs nNOS *P=0.03; PV vs ChAT P=0.28). **e**, Pie-charts displaying a comparison of eYFP$^+$/neuron subclass labeled markers in NAc core and shell; putative D1, D2-MSNs, and PV-interneurons had elevated anterograde labeling, suggesting strong synaptic connections with PVT. Data are presented as mean values +/- SEM.

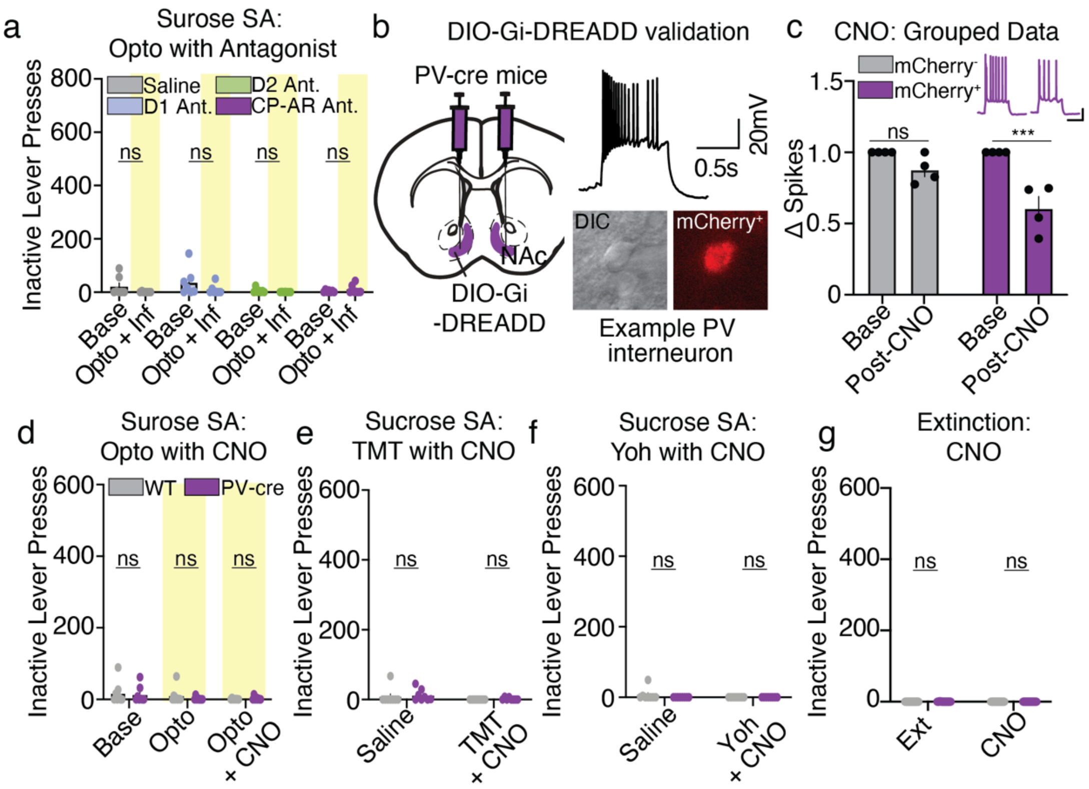

Supplementary Figure 6. Selective DREADD-mediated inhibition of PV-interneurons, and inactive lever responding during pharmacological and chemogenetic manipulations.

a, Inactive lever pressing remained unchanged despite optogenetic activation of PVT→NAc neurons with or without intra-NAc infusions of antagonists for D1 receptors, D2 receptors, and CP-AMPARs (*n*=7 mice/group; repeated-measures two-way ANOVA, *F*-values<3.61, *P*-values>0.07). **b**, Viral strategy (left) and example PV interneuron (right) waveform and expression. **c**, Waveforms (scale: 20mV/0.5s) and grouped data (*n*=4 cells, 2-4 mice/group) revealed that mCherry^{+} neurons spiked significantly less than neighboring mCherry^{-} neurons following bath application of CNO (repeated-measures two-way ANOVA, time x group interaction: $F_{1,12}$=7.68, P<0.01; Sidak's post-hoc: mCherry^{+} ****P*=0.0002). **d-g**, Inactive lever pressing remained unchanged despite optogenetic manipulation of PVT→NAc neurons with or without chemogenetic inhibition of PV interneurons through intra-NAc infusions of CNO (**d**, Opto: *n*=8 mice/group; *F*-values<2.33, *P*-values>0.13; **e**, TMT: *n*=8 mice/group; repeated-measures two-way ANOVA, *F*-values<3.19, *P*-values>0.09; **f**, yohimbine: *n*=8 mice/group; repeated-measures two-way ANOVA, *F*-values<1.19, *P*-values>0.29; **g**, extinction: *n*=7 mice/group; repeated-measures two-way ANOVA, *F*-values<2.29, *P*-values>0.12). SA, self-administration; Base, baseline; Opto, optogenetics; Inf, infusion; Ant., antagonist; Yoh, yohimbine; Ext, extinction. Bar graphs are presented as mean values +/- SEM. Source data are provided as a Source Data file.

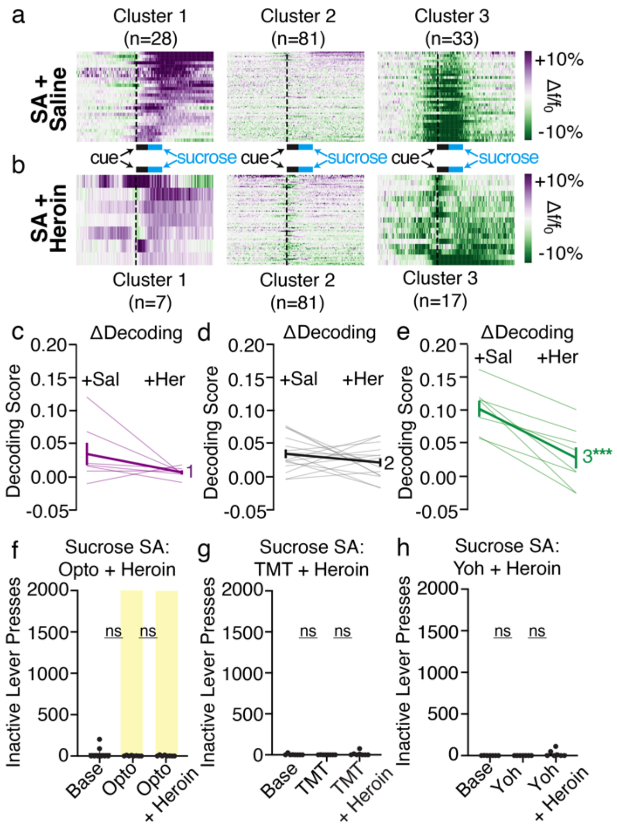

Supplementary Figure 7. Heroin administration modulates PVT-NAc neuronal ensemble dynamics, but not inactive lever pressing.

a-**b**, Heatmaps for each neuronal ensemble during sucrose self-administration following an intraperitoneal injection of saline (**a**; *n*=142 cells/4 mice) or heroin (**b**; *n*=105 cells/4 mice). **c-e**, Decoding scores for tracked cells in ensemble #1 (**c**), #2 (**d**), and #3 (**e**), show that heroin significantly reduces decoding for inhibited ensemble #3 (two-tailed t-test, t_7=5.89, *P*=0.001), but not other ensembles (two-tailed t-tests, *t*-scores<1.6, *P*-values>0.15). **f-h**, Inactive lever pressing remained unchanged across all conditions with or without a simultaneous systemic injection of heroin (**f**, Opto: *n*=8 mice/group; one-way ANOVA, $F_{2,21}$=1.59, *P*=0.23; **g**, TMT: *n*=8 mice/group; one-way ANOVA, $F_{2,21}$=1.22, *P*=0.32; **h**, yohimbine: *n*=7 mice/group; one-way ANOVA, $F_{2,18}$=2.25, *P*=0.13). SA, self-administration; Base, baseline; Opto, optogenetics, Yoh, yohimbine. Bar and line graphs are presented as mean values +/- SEM. Source data are provided as a Source Data file.

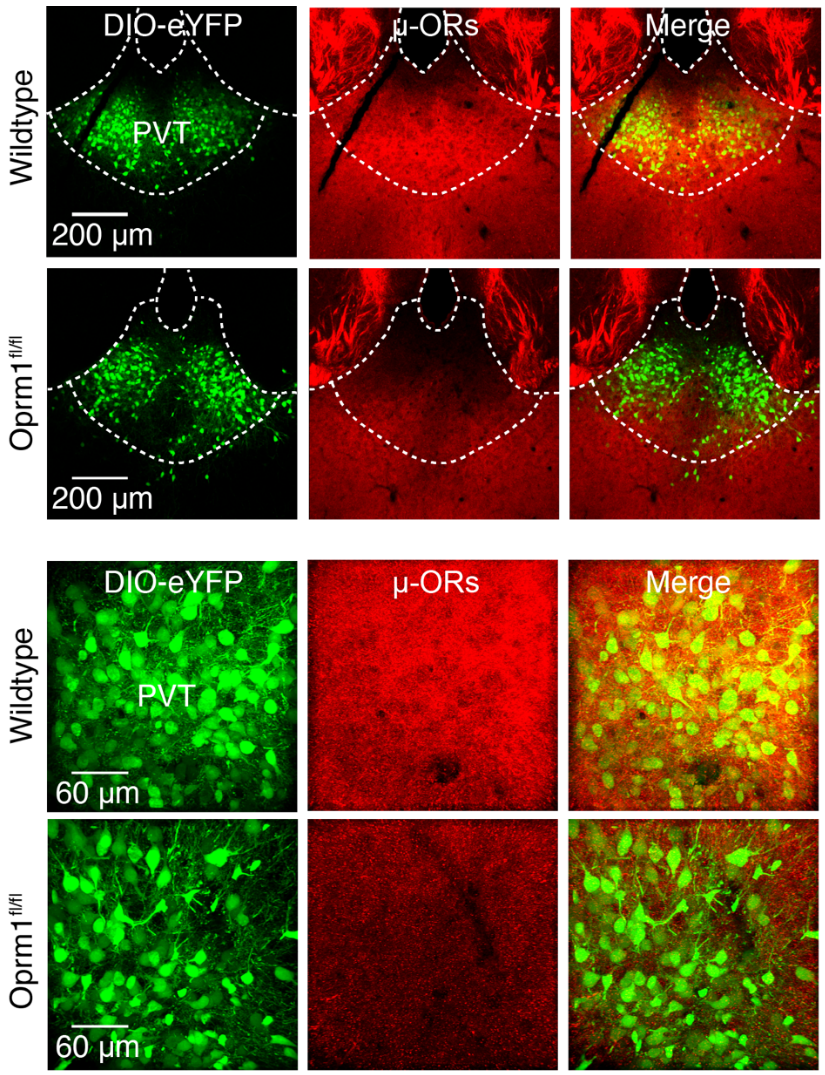

Supplementary Figure 8. Immunohistochemistry of PVT→NAc μ-opioid receptor expression in WT and *Oprm1$^{fl/fl}$* mice.

Zoomed-out (top rows) and zoomed-in (bottom rows) images of PVT→NAc μ-OR expression in WT and *Oprm1$^{fl/fl}$* mice following Cre-dependent knockout.

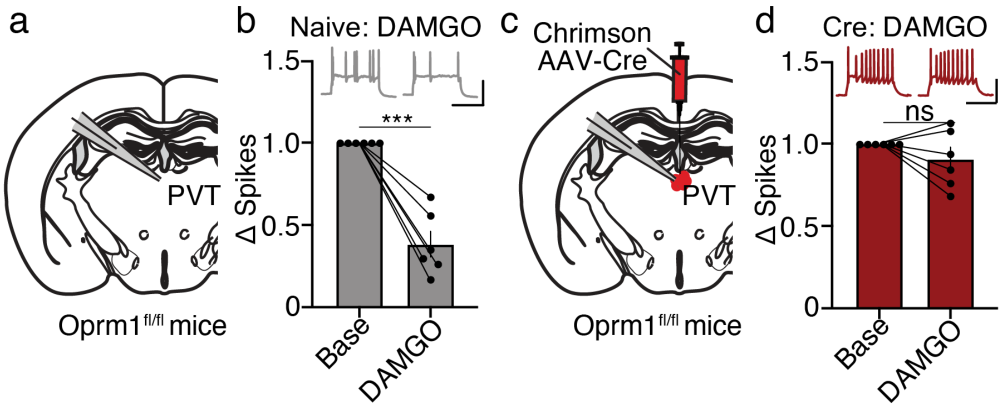

Supplementary Figure 9. DAMGO-induced suppression of PVT firing rates is prevented by knockout of PVT μ-opioid receptors.

a, Electrophysiological strategy for recording PVT neurons in naïve *Oprm1[fl/fl]* mice. **b**, Example waveforms (scale: 50mV/0.5s) and grouped data from patched PVT neurons show a decrease in spiking following DAMGO (n=6 cells, 2 mice; two-tailed t-test, t_5=7.89, P=0.001). **c**, Viral strategy for Cre-dependent knockout of PVT μ-ORs in *Oprm1[fl/fl]* mice. **d**, Example waveforms (scale: 50mV/0.5s) and grouped data show knockout of PVT μ-ORs prevents DAMGO-induced decrease of spiking (n=6 cells, 4 mice; two-tailed t-test, t_5=1.29, P=0.26). μ-OR, μ-opioid receptor; Base, Baseline. Bar graphs are presented as mean values +/- SEM. Source data are provided as a Source Data file.

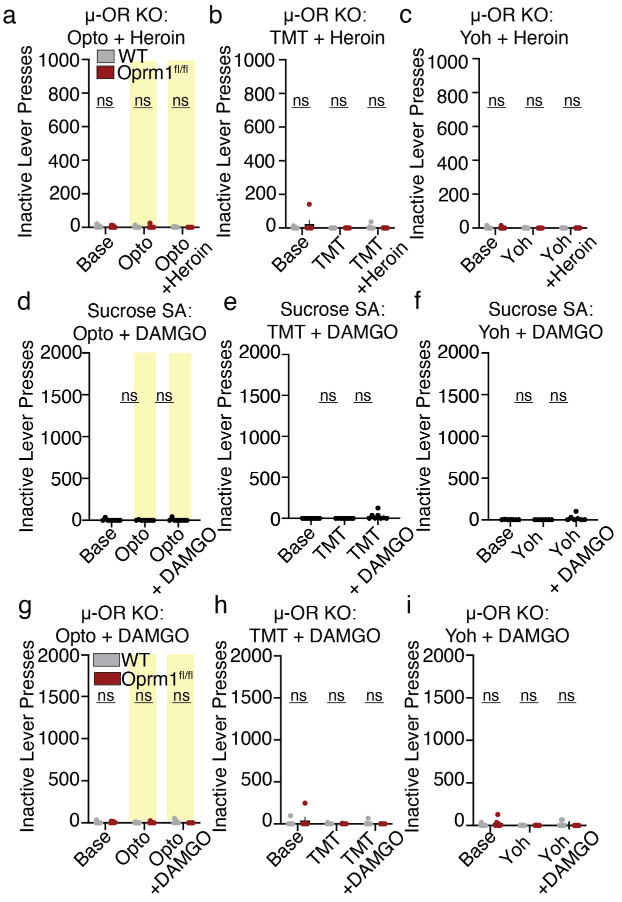

Supplementary Figure 10. Inactive lever pressing remains low after intraperitoneal heroin injection or intracranial DAMGO infusion in WT and *Oprm1$^{fl/fl}$* mice.

a-c, Inactive lever pressing remained unchanged across all conditions and groups with or without a simultaneous systemic injection of heroin (**a**, μ-OR KO Opto: *n*=6 *Oprm1$^{fl/fl}$*, 8 WT mice; repeated-measures two-way ANOVA, $F_{2,24}$=0.54, *P*=0.59; **b**, μ-OR KO TMT: *n*=6 *Oprm1$^{fl/fl}$*, 8 WT mice; repeated-measures two-way ANOVA, $F_{2,24}$=1.35, *P*= 0.28; **c**, μ-OR KO yohimbine: *n*=6 *Oprm1$^{fl/fl}$*, 8 WT mice; repeated-measures two-way ANOVA, $F_{2,24}$=0.15, *P*=0.86). **d-i**, Inactive lever pressing remained unchanged across all conditions and groups with or without an intra-NAc infusion of DAMGO (**d**, Opto: *n*=8 mice/group; one-way ANOVA, $F_{2,21}$=0.32, *P*=0.73; **e**, TMT: *n*=8 mice/group; one-way ANOVA, $F_{2,21}$=2.83, *P*=0.08; **f**, yohimbine: *n*=8 mice/group; one-way ANOVA, $F_{2,18}$=2.09, *P*=0.15; **g**, μ-OR KO Opto: *n*=6 *Oprm1$^{fl/fl}$*, 8 WT mice; repeated-measures two-way ANOVA, $F_{2,24}$=1.62, *P*=0.23; **h**, μ-OR KO TMT: *n*=6 *Oprm1$^{fl/fl}$*, 8 WT mice; repeated-measures two-way ANOVA, $F_{2,24}$=0.82, *P*= 0.45; **i**, μ-OR KO yohimbine: *n*=6 *Oprm1$^{fl/fl}$*, 8 WT mice; repeated-measures two-way ANOVA, $F_{2,24}$=2.63, *P*=0.09). μ-OR, μ-opioid receptor; KO, knockout; WT, wild-type; Yoh, yohimbine; SA, self-administration. Bar graphs are presented as mean values +/- SEM. Source data are provided as a Source Data file.

Supplementary Table 1. Primary resources for immunohistochemistry.

Primary antisera	Host species	Concentration	Source	RRID (AB_)	Immunogen	Secondary antisera
Choline acetyltransferase	Mouse	1:1000	Millipore, AMAB91130	2665812	Peptide sequence	Anti-mouse 647
Parvalbumin	Mouse	1:1000	Millipore, MAB1572	2174013	Parvalbumin purified from frog muscle	Anti-mouse 647
Pre-pro Enkephalin	Rabbit	1:200	Neuromics, RA14124	2532106	Peptide sequence	Anti-rabbit 647
nNOS	Rabbit	1:1000	Millipore, AB5380	91824	Recombinant human nNOS	Anti-rabbit 647
μ-Opioid Receptor	Rabbit	1:500	Abcam, AB134054	N/A	Peptide sequence	Anti-rabbit 647
NeuN	Mouse	1:1000	Millipore, MAB377	2298772	Purified cell nuclei	Anti-mouse 647
Cre	Mouse	1:1000	Millipore MAB3120	2085748	Cre-recombinase fusion protein	Anti-mouse 647
