# Supplementary material for: An opioid-gated thalamoaccumbal circuit for the suppression of reward seeking in mice
Source: Nat Commun. 2022 Nov 11;13:6865. doi: 10.1038/s41467-022-34517-w (PMC9652456; doi:10.1038/s41467-022-34517-w)
Supplement: Supplementary file 3 — Reporting Summary [file 41467_2022_34517_MOESM3_ESM.pdf]

nature portfolio

Corresponding author(s): James M. Otis

Last updated by author(s): 10/18/2022

Reporting Summary

Nature Portfolio wishes to improve the reproducibility of the work that we publish. This form provides structure for consistency and transparency in reporting. For further information on Nature Portfolio policies, see our Editorial Policies and the Editorial Policy Checklist.

Statistics

For all statistical analyses, confirm that the following items are present in the figure legend, table legend, main text, or Methods section.

n/a	Confirmed	
☐	☒	The exact sample size (*n*) for each experimental group/condition, given as a discrete number and unit of measurement
☐	☒	A statement on whether measurements were taken from distinct samples or whether the same sample was measured repeatedly
☐	☒	The statistical test(s) used AND whether they are one- or two-sided<br>*Only common tests should be described solely by name; describe more complex techniques in the Methods section.*
☐	☒	A description of all covariates tested
☐	☒	A description of any assumptions or corrections, such as tests of normality and adjustment for multiple comparisons
☐	☒	A full description of the statistical parameters including central tendency (e.g. means) or other basic estimates (e.g. regression coefficient) AND variation (e.g. standard deviation) or associated estimates of uncertainty (e.g. confidence intervals)
☐	☒	For null hypothesis testing, the test statistic (e.g. *F*, *t*, *r*) with confidence intervals, effect sizes, degrees of freedom and *P* value noted<br>*Give P values as exact values whenever suitable.*
☒	☐	For Bayesian analysis, information on the choice of priors and Markov chain Monte Carlo settings
☒	☐	For hierarchical and complex designs, identification of the appropriate level for tests and full reporting of outcomes
☒	☐	Estimates of effect sizes (e.g. Cohen's *d*, Pearson's *r*), indicating how they were calculated

Our web collection on statistics for biologists contains articles on many of the points above.

Software and code

Policy information about availability of computer code

Data collection	Parameters for behavioral sessions were set using MATLAB that controlled an Arduino and associated electronics. Data were recorded in MATLAB code, and transfered to Python for organization and analysis. Imaging data were acquired using PrarieView software (Bruker Imaging).
Data analysis	Behavioral data were extracted using Python (v2.7), and analyzed and graphed using GraphPad PRISM (v8), and illustrated using Adobe Illustrator (v26). Imaging data were converted into hdf5 format, and motion corrected using SIMA (v1.3.2). Next, imaging data was analyzed using a custom-written Python code. Within the python code, a Principal components analysis was used to inform the Scikit-learn function sklearn.cluster.SpectralClustering, a spectral clustering algorithm. For, decoding analyses, a binary decoder was used the Scikit-learn functions sklearn.discriminant_analysis, sklearn.smv, and sklearn.decomposition. For confocal imaging analyses, the Imaris (v9.0) "spot" tool was used to mark and identify individual cells, wherein cell count or or intensity values could be collected. For patch clamp electrophysiological data, Clampfit (pClamp v11) was used to to analyze recordings of spiking or synaptic currents.

For manuscripts utilizing custom algorithms or software that are central to the research but not yet described in published literature, software must be made available to editors and reviewers. We strongly encourage code deposition in a community repository (e.g. GitHub). See the Nature Portfolio guidelines for submitting code & software for further information.

Data

Policy information about availability of data

All manuscripts must include a data availability statement. This statement should provide the following information, where applicable:

- Accession codes, unique identifiers, or web links for publicly available datasets
- A description of any restrictions on data availability
- For clinical datasets or third party data, please ensure that the statement adheres to our policy

All behavioral and electrophysiological data are available at https://github.com/jimotis. Two-photon imaging datasets are available upon reasonable request.

Field-specific reporting

Please select the one below that is the best fit for your research. If you are not sure, read the appropriate sections before making your selection.

☐ Life sciences ☒ Behavioural & social sciences ☐ Ecological, evolutionary & environmental sciences

For a reference copy of the document with all sections, see nature.com/documents/nr-reporting-summary-flat.pdf

Behavioural & social sciences study design

All studies must disclose on these points even when the disclosure is negative.

Study description	This study measures neuronal activity and behavioral output following different treatments, all data is quantitative.
Research sample	Adult (8-24 weeks old) male and female mice. Mice were chosen as transgenic lines are available in reward-behaving animals.
Sampling strategy	Mice were assigned to groups randomly, and sizes for each study were predetermined based on our previous studies and similar work.
Data collection	Behavioral data were collected using a custom-built behavioral chamber equipped with an infusion pump, operant levers, speaker, head-fixation station, Arduino board, lasers (green and blue), and laptop. Lever responses and infusions were recorded with a custom-written MATLAB code. Real-time place preference was conducted using a standard 2-chamber conditioning apparatus. Imaging data was collected using a two-photon microscope (Bruker Nano Inc) equipped with a tunable InSight DeepSee laser (Spectra Physics, laser set to 920nm, ~100fs pulse width), resonant scanning mirrors (~30Hz framerate), a 20X air objective (Olympus, LCPLN20XIR, 0.45NA, 8.3mm working distance), and GaAsP photodetectors. Imaging data were acquired using Prarieview software. Immunohistochemistry images were collected with a confocal microscope (Leica SP8). A select few researchers handled mice in each experiment, and were blinded to experimental conditions when possible.
Timing	Data collection began in June, 2020 and has continued until present.
Data exclusions	Exclusion criteria were predetermined as 1) if a mouse was unable to extinguish lever pressing during extinction training for 25 consecutive days, or 2) if operant lever responding for a particular day was 3 standard deviations above or below the mean number of lever presses for that day. Only one day was excluded, which was during sucrose acquisition.
Non-participation	Mice who were euthanized (e.g., because of not recovering from surgery) or died unexpectedly did not participate or were removed from the study (total of 8 mice). One mouse brain was lost during RNAscope tissue preparation.
Randomization	Mice were randomly allocated into groups., with a roughly equal number of males and females in each group. Testing days were psuedo-randomly ordered when possible.

Reporting for specific materials, systems and methods

We require information from authors about some types of materials, experimental systems and methods used in many studies. Here, indicate whether each material, system or method listed is relevant to your study. If you are not sure if a list item applies to your research, read the appropriate section before selecting a response.

March 2021

Materials & experimental systems

n/a	Involved in the study
☐	☒ Antibodies
☒	☐ Eukaryotic cell lines
☒	☐ Palaeontology and archaeology
☐	☒ Animals and other organisms
☒	☐ Human research participants
☒	☐ Clinical data
☒	☐ Dual use research of concern

Methods

n/a	Involved in the study
☒	☐ ChIP-seq
☒	☐ Flow cytometry
☒	☐ MRI-based neuroimaging

Antibodies

Antibodies used

Choline acetyltransferase: Millipore / Cat. No. AMAB91130 / CL3173, Mouse monoclonal / RRID (AB_2665812);
Parvalbumin: Millipore / Cat. No. MAB1572 / PARV-19, Mouse monoclonal / RRID (AB_2174013)
Pre-pro enkephalin: Neuromics / Cat. No. RA14124 / Rabbit polyclonal / RRID (AB_2532106)
nNOS: Millipore / Cat. No. AB5380 / Rabbit polyclonal / RRID (AB_91824)
μ-Opioid receptor: Abcam / Cat. No. AB134054 / Rabbit monoclonal / UMB3
NeUN: Millipore / Cat. No. MAB377 / A60, Mouse monoclonal / RRID (AB_2298772)
Cre: Millipore / Cat. No. MAB3120 / 2D8, Mouse monoclonal / RRID (AB_2095748)
Anti-Mouse 647: Invitrogen / Cat No. A-21236 / Polyclonal Goat anti-mouse IgG / RRID (AB_2535805)

Validation

Choline acetyltransferase: From manufacturer: "Orthogonal validation of protein expression using IHC by comparison to data of corresponding target in high and low expression tissues". References: https://www.proteinatlas.org/ENSG00000070748-CHAT/tissue/primary+data; https:// www.proteinatlas.org/ENSG00000070748-CHAT/subcellular; PMID: 33239269

Parvalbumin: From manufacturer: "Independent validation by the NYU Lagone was performed for: IHC." References: PMID: 17072835; PMID: 17990269; PMID: 19480001

Pre-pro enkephalin: References: PMID: 30811995; PMID: 2082261; PMID: 28521136

nNOS: References: PMID: 2812301; PMID: 29620525; PMID: 29858554

μ-Opioid receptor: References: PMID: 24413699, PMID: 32451910, PMID: 20851148

NeUN: From manufacturer: "Independent validation by the NYU Lagone was performed for: IHC." References: PMID: 16736475; PMID: 17335037; PMID: 17444497

Cre: References: PMID: 25643298; PMID: 24886120; PMID: 22745484

Animals and other organisms

Policy information about studies involving animals; ARRIVE guidelines recommended for reporting animal research

Laboratory animals

Adult male and female C57BL6/J wild-type, PV-Cre (B6.Cg-Pvalbtml.l(cre)Aibs/J, Strain #012358), D2-Cre (Drd2, line ER44, RRIDMMRRC_017263-UCD), B6129SF2/J WT (Jax Strain #101045), and Oprmlfl/fl (B6.129-Oprmltml.lCgrf/KffJ, Strain #030074) mice, all aged greater than 8 weeks and at least 20g prior to study onset. Colony was maintained at ~20° Celcius / 50% humidity.

Wild animals

Study did not involve wild animals. .

Field-collected samples

Study did not involve field-collected samples.

Ethics oversight

All experiments were approved by the Institutional Animal Care and Use Committee (IACUC) at the Medical University of South Carolina in accordance with the NIH-adopted Guide for the Care and Use of Laboratory Animals

Note that full information on the approval of the study protocol must also be provided in the manuscript.
